# Natural Products in the Treatment of Retinopathy of Prematurity: Exploring Therapeutic Potentials

**DOI:** 10.3390/ijms25158461

**Published:** 2024-08-02

**Authors:** Jong-Ik Heo, Juhee Ryu

**Affiliations:** College of Pharmacy and Research Institute of Pharmaceutical Sciences, Kyungpook National University, Daegu 41566, Republic of Korea; ik0412@knu.ac.kr

**Keywords:** natural products, angiogenesis, retinopathy of prematurity, retinal vascular disease

## Abstract

Retinopathy of prematurity (ROP) is a vascular disorder affecting the retinas of preterm infants. This condition arises when preterm infants in incubators are exposed to high oxygen levels, leading to oxidative stress, inflammatory responses, and a downregulation of vascular endothelial growth factors, which causes the loss of retinal microvascular capillaries. Upon returning to room air, the upregulation of vascular growth factors results in abnormal vascular growth of retinal endothelial cells. Without appropriate intervention, ROP can progress to blindness. The prevalence of ROP has risen, making it a significant cause of childhood blindness. Current treatments, such as laser therapy and various pharmacologic approaches, are limited by their potential for severe adverse effects. Therefore, a deeper understanding of ROP’s pathophysiology and the development of innovative treatments are imperative. Natural products from plants, fungi, bacteria, and marine organisms have shown promise in treating various diseases and have gained attention in ROP research due to their minimal side effects and wide-ranging beneficial properties. This review discusses the roles and mechanisms of natural products that hold potential as therapeutic agents in ROP management.

## 1. Retinopathy of Prematurity

Retinopathy of prematurity (ROP) is a vascular disease that can affect the retinas of immature infants [1]. ROP can occur in preterm infants with a gestational age of 30 weeks or less. The risk of developing ROP is higher in infants born at 28 weeks gestation or earlier, or those with a very low birth weight of 1.5 kg or less, as the blood vessels in the retina are still under development at this stage [2,3,4]. The age-standardized rate (ASR) of global prevalence of ROP indicates 86.4 cases of vision loss per 100,000 population, with 31.6 cases attributed to ROP among those under 20 years old. The ASR for blindness and vision loss due to ROP has shown a significant increasing trend since 1990 [5]. Adults who were preterm infants requiring treatment for ROP often experience reduced visual acuity and diminished vision-related quality of life [6]. If not treated promptly, ROP can lead to permanent blindness [7,8].

**Figure 1:**
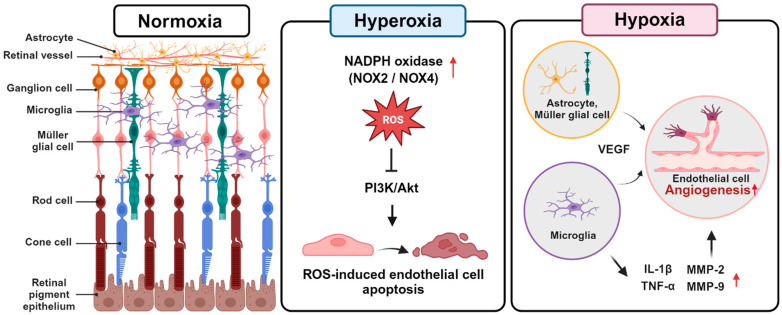
Cellular mechanisms involved in the development of retinopathy of prematurity (ROP). During hyperoxic conditions, phase 1, nitro-oxidative stress induces apoptosis in retinal capillary endothelial cells by disrupting the PI3K/Akt signaling pathway. During hypoxic conditions, phase 2, astrocytes and Müller glial cells secrete VEGF, while microglia secrete IL-1β, TNF-α, and metalloproteinases, promoting neovascularization by endothelial cells.

The progression of ROP involves two phases: Phase I and phase II. During phase I, ROP develops in response to hyperoxia due to supplemental oxygen in the incubator. In this vaso-obliterative phase, there is an interruption and delay in retinal vascular growth, accompanied by microvascular degeneration [9]. During this phase, the supply of oxygen downregulates vascular endothelial growth factor (VEGF), hypoxia-inducible factor (HIF)-1, insulin-like growth factor-1 (IGF-1), and erythropoietin (EPO), while oxidative stress is increased due to the upregulation of NADPH oxidase such as NOX2 and NOX4 and the uncoupling of endothelial nitric oxide synthase (eNOS) [10,11,12]. Preterm infants, with a relative deficiency in antioxidant systems, experience increased damage from elevated reactive oxygen species (ROS) [13]. Hyperoxia-induced nitro-oxidative stress in retinal endothelial cells leads to retinal capillary endothelial cell apoptosis by interfering with the phosphatidylinositol 3-kinase/protein kinase B (PI3K/Akt) signaling pathway [14] (Figure 1).

Subsequently, in phase II, when infants are returned to normoxia—a relatively hypoxic state compared to the incubator—retinal neovascularization (RNV) occurs [15,16]. Hypoxic conditions stimulate the expression of HIF-1α, which, in turn, promotes retinal angiogenesis by regulating the expression of proangiogenic factors such as VEGF [17]. VEGF is initially secreted by astrocytes and later expressed by Müller glial cells, promoting the formation of retinal blood vessels through the migration of vascular endothelial cells [18]. Additionally, VEGFR-2 is present on retinal endothelial cells and responds to VEGF stimulation, leading to signaling that directs endothelial cell division and growth [19]. Increased expression of VEGFR-2 can lead to abnormal vascular formation [20], which can ultimately lead to vision loss. Moreover, retinal microglia produce significant amounts of inflammatory factors such as interleukin (IL)-1β, tumor necrosis factor (TNF)-α, and matrix metalloproteinase (MMP)-2 and -9 [21,22] (Figure 1).

Several factors are implicated in ROP. During the hypoxic phase, key molecular players such as VEGF, HIF-1α, angiopoietin 2, EPO, and IGF-1 are upregulated [23,24,25,26,27]. Numerous signaling pathways, including those mediated by VEGF, extracellular signal-regulated kinase (ERK), PI3K/Akt, and platelet-derived growth factor β (PDGF-β), contribute to the development of RNV [10,28]. Specifically, in the VEGF signaling pathway, hypoxic conditions lead to an upregulation of HIF-1α, which subsequently increases VEGF expression and drives RNV [29]. VEGF-A plays a critical role in both physiological and pathological angiogenesis. Among the various VEGF receptors, VEGFR2 predominantly facilitates the proliferation and migration of endothelial cells. Activation of VEGFR-2 triggers downstream signaling pathways such as ERK and PI3K/Akt. The ERK pathway, a component of the mitogen-activated protein kinase (MAPK) family, is essential for cellular processes including division, differentiation, and survival, and it supports RNV by enhancing endothelial cell proliferation and migration [30]. The PI3K/Akt pathway, initiated by PI3K activation, leads to the production of phosphatidylinositol-3,4,5-trisphosphate (PIP3), which recruits and activates Akt. This activation promotes the proliferation and survival of endothelial cells, further contributing to RNV. Additionally, the PI3K/Akt pathway can upregulate the expression of eNOS, resulting in vasodilation and further progression of retinal angiogenesis [31,32,33]. PDGF-β plays a crucial role in attracting pericytes and vascular smooth muscle cells to stabilize blood vessels. However, downregulation of PDGF-β can lead to pericyte loss, abnormal vasodilation, and increased VEGF expression [28].

## 2. Natural Compounds

Natural compounds, derived from various sources such as plants and marine microbes, have historically been a primary source of medicinal agents. Penicillin, for instance, a groundbreaking antibiotic, was isolated from the microorganism *Penicillium notatum* [34]. Similarly, aspirin, originally extracted from the salicin in willow trees, serves as a widely used analgesic and anti-inflammatory agent [35]. Furthermore, digoxin, a cardiotonic glycoside from *Digitalis purpurea*, enhances cardiac contractility in patients with heart failure and is recognized for its enduring therapeutic effects [36]. Lastly, cyclosporine, a cyclic polypeptide produced by the fungus *Tolypocladium inflatum*, has been developed as an ophthalmic emulsion to enhance tear production and has been approved by the FDA for the treatment of chronic dry eye [37].

In the 20th century, natural compounds comprised a significant portion of all approved drugs. However, recent advancements have seen a shift toward synthetic drugs that target specific proteins, such as ranibizumab and aflibercept. Despite this, the role of natural compounds as adjunct therapies to first-line treatments continues to be of interest due to their potential to enhance synergistic effects. Techniques such as methylation, glycosylation, acetylation, and conjugation are utilized to modify the structure of natural compounds, aiming to optimize their absorption, distribution, metabolism, excretion, and minimize toxicity [38,39]. Therefore, natural compounds remain a compelling source for novel drug development. Several natural products, including sinecatechins (Veregen^®^) and crofelemer (Mytesia™), have received FDA approval [40,41]. Moreover, numerous compounds such as resveratrol, inositol, quercetin, and curcumin are currently undergoing clinical trials [42,43].

Various natural compounds have been found to have beneficial effects on ocular diseases. Curcumin, derived from turmeric, and resveratrol, derived from grapes, berries, and peanuts, exhibit antioxidant and anti-inflammatory properties, which are beneficial in treating diabetic retinopathy (DR) [44,45,46]. Additionally, berberine, obtained from barberry and goldenseal, has demonstrated anti-inflammatory and anti-angiogenic effects [47,48]. In the context of age-related macular degeneration (AMD) and cataracts, lutein and zeaxanthin (L/Z), sourced from green leafy vegetables, corn, and eggs, are known for their antioxidant properties [49,50]. Oral antioxidant and L/Z supplementation have been shown to slow the progression of geographic atrophy in patients with AMD, highlighting its potential in preserving central macular function [51]. Additionally, astaxanthin, extracted from salmon, krill, and algae, has shown antioxidant and anti-inflammatory effects in AMD [52,53]. Water-soluble ingredients derived from marine fish species such as *Decapterus tabl* have shown anti-angiogenic properties by inhibiting the expression of HIF and VEGF in vitro and reducing RNV in an oxygen-induced retinopathy (OIR) in vivo model [54]. Furthermore, marine extracts from fish species, such as *Decaperus tabl* and *Depapterus muroadsi*, and their active ingredient taurine, have been shown to reduce HIF-1α expression under hypoxic conditions in vitro and choroidal neovascularization and fibrosis in an in vivo AMD model [55]. For glaucoma, Ginkgo biloba extract, derived from the Ginkgo biloba tree, has been shown to enhance blood flow and exhibit antioxidant properties [56].

Natural compounds can be classified based on their chemical structures. Various structures, including polyphenols, carotenoids, and alkaloids, have been effective in treating ocular diseases [57]. Polyphenols are subdivided into flavonoid and non-flavonoid compounds. Quercetin and luteolin, flavonoids, have been shown to reduce oxidative stress and inflammation in AMD. Among non-flavonoids, curcumin is identified as a curcuminoid, and resveratrol as a stilbene; both have been effective in DR and AMD [58,59]. Astaxanthin, lutein, and zeaxanthin, which are carotenoids, have been found effective in reducing oxidative stress in AMD [50,53]. Berberine, an alkaloid, has shown efficacy in DR [60].

In this review, we included the articles investigating the effects of natural compounds in the retinopathy of the prematurity model. We utilized PubMed to collect relevant articles for our research topic using disease-related key terms such as retinopathy of prematurity and oxygen-induced retinopathy and therapeutic target-related key terms such as natural compounds or natural products, along with source-related key term such as plants, vegetables, fungi, marine organisms. Then, articles were manually reviewed, and the papers that included experiments assessing the therapeutic effects of active compounds from natural products in cellular or animal models were included. Chinese herbal formulas were excluded since they could contain multiple ingredients; therefore, evaluating the effects of a single active compound may be difficult.

## 3. Natural Compounds in the Modulation of ROP

**Figure 2:**
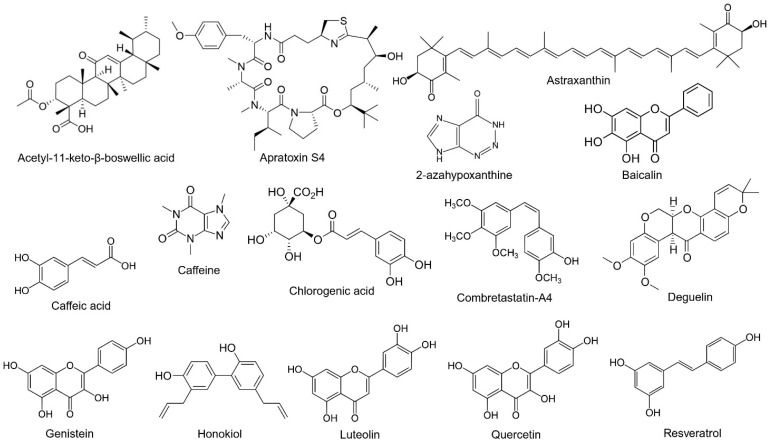
Structures of natural compounds. Chemical structures of selected natural products included in the review are shown.

**Figure 3:**
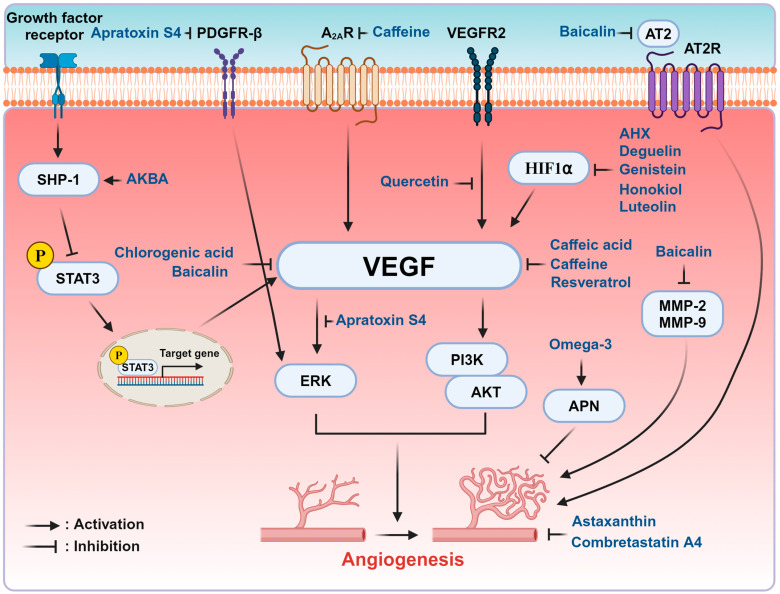
Mechanisms of Natural Products in the progression of ROP. The Regulatory mechanisms of natural compounds discussed in this article are demonstrated at the cellular level. Natural compounds act as inhibitors of angiogenesis by regulating angiogenesis-related receptors or engaging various signaling pathways. Activation is indicated by pointed arrows, while inhibition is represented by T-bars. Abbreviations: AHX, 2-Azahypoxanthine; AKBA, acetyl-11-keto-b-boswellic acid; A_2A_R, adenosine A2A receptor; APN, adiponectin; AKT, protein kinase B; AT2R, angiotensin II receptor; ERK, extracellular signal-regulated kinase; HIF1α, hypoxia-inducible factor 1α; MMP, matrix metalloproteinase; PDGFR-β, platelet-derived growth factor β; PI3K, phosphatidylinositol 3-kinase; SHP-1, Src homology region 2 domain-containing phosphatase 1; STAT3, signal transducer and activator of transcription 3 VEGFR2, vascular endothelial growth factor receptor 2.

Various natural products from plants, marine microbes, fruits, and vegetables have been identified as regulators of ROP (Table 1). Many of these compounds exhibit anti-angiogenic properties, with some also demonstrating antioxidant effects. In vitro studies utilizing human retinal microvascular endothelial cells (HRMECs), human retinal microvascular endothelial cells (HUVECs), retinal pigment epithelial cells, and human retinal pericytes (HRPCs) have established a cellular model for ROP, with endothelial cells being the most frequently used. VEGF stimulation or hypoxic conditions are commonly employed to simulate ROP in cellular models [61,62]. To simulate ROP in animals, the OIR model using mice was commonly employed along with persistent RNV in rabbits [63,64]. In brief, one-week-old mice are exposed to 75% oxygen for 5 days from P7 to P12 and returned to room air [64]. In rabbits, intravitreal injection of DL-alpha-aminoadipic acid (DL-AAA) induces damage to retinal Müller glial cells, resulting in persistent RNV that occurs after 48 weeks post-injection [63]. Flavonoids are among the most frequently reported natural products that regulate ROP. The structures of selected natural products are presented in Figure 2. Mechanisms of natural compounds that may have therapeutic potential for the treatment of ROP are depicted in Figure 3.

2-Azahypoxanthine (AHX), a natural compound derived from mushroom-forming fungi such as *Lepista sordida*, has demonstrated anti-angiogenic potential in OIR models [65]. AHX inhibited hypoxia-activated genes, including VEGF, under hypoxic conditions in CoCl_2_-induced ARPE-19 and 661 W cell lines. Furthermore, oral administration of AHX inhibited retinal neovascular tufts in an OIR mouse model. AHX suppresses HIF-1α activation and the expression of VEGF without inhibiting HIF-1α stabilization. This study shows that AHX can serve as a potential treatment for ROP through inhibition of HIF-1α and pathological RNV.

Acetyl-11-keto-β-boswellic acid (AKBA), a pentacyclic triterpene sourced from *Boswellia serrata*, has been studied for its effects in the OIR model [66]. Prior research has shown that Src homology region 2 domain-containing phosphatase 1 (SHP-1) can dephosphorylate the signal transducer and activator of transcription 3 (STAT3) [67], a process that, when inhibited, reduces the transcription of VEGF [68]. Lulli et al. [66] demonstrated that AKBA administration upregulated SHP-1 expression while downregulating phosphorylated STAT3 and VEGF in the retinas of OIR mice, thereby inhibiting the VEGF signaling pathway through the SHP-1/STAT3/VEGF axis. Additionally, AKBA was shown to suppress proliferation, tube formation, and migration in VEGF-stimulated HRMECs, highlighting its potential role in inhibiting pathological retinal angiogenesis.

Apratoxin S4, a cyclodepsipeptide derived from marine cyanobacterium *Lyngbya majuscula*, also exhibits anti-angiogenic properties [69]. It has been shown to inhibit proliferation and migration in VEGF-stimulated human retinal endothelial cells (HRECs) and reduce angiogenic signaling pathways such as those involving VEGF and Erk. Furthermore, apratoxin S4 inhibited migration and PDGFR-β in human retinal pericytes and suppressed vessel sprouting in VEGF-induced aortic rings and pathological retinal angiogenesis in OIR mouse retinas. These findings suggest that apratoxin S4 may suppress RNV by regulating multiple angiogenic signaling pathways.

Astaxanthin, a carotenoid found in various marine organisms, including microalgae, salmon, krill, and shrimp, was reported to have anti-angiogenic and antiproliferative effects [70]. Compared to control mice, mice injected with intravitreal and intraperitoneal astaxanthin had significant reductions in RNV. In particular, intravitreal injection of astaxanthin reduced RNV more effectively than intraperitoneal injection. Administration of astaxanthin inhibited mitochondrial damage in retinal cells in electron microscopy. High doses of astaxanthin decreased the number of apoptotic cells without disrupting retinal vascular development and causing toxicity. Since the anti-angiogenic and antiapoptotic effects of astaxanthin have been found, further studies can explore the optimal dose and route of administration.

Baicalin, a flavonoid isolated from *Scutellaria baicalensis*, demonstrated anti-angiogenic properties in an ROP mouse model [71]. Both high-dose (10 mg/kg) and low-dose (1 mg/kg) baicalin injections intraperitoneally suppressed the development of avascular areas and neovascular tufts and lumens significantly. Particularly, high-dose baicalin significantly reduced the expression of VEGF, angiotensin II, and MMP-2 and -9 in retinas. Thus, this study revealed that the intraperitoneal administration of baicalin effectively inhibits RNV. Additionally, alternative routes of administration, such as oral or intravitreal injection, along with precise pharmacokinetic and mechanistic studies, can be considered to assess its clinical potential.

Caffeic acid, a phenolic compound found in coffee, vegetables, and fruits, exhibited anti-angiogenic and antioxidant effects in ROP [72]. It reduced cell proliferation, migration, and tube formation in VEGF-stimulated HRMECs in a dose-dependent manner. Administration of caffeic acid decreased ROS levels and VEGF expression in H_2_O_2_-treated HRMECs, highlighting its antioxidant properties. Furthermore, intravitreal injection of caffeic acid prevented the development of vascular lumens in OIR mice without inducing structural changes or inflammation in the retinal layers.

Caffeine, a trimethylxanthine derived from coffee and tea, effectively suppresses vessel loss during the hyperoxic phase and inhibits abnormal vessel development during the hypoxia phase in the OIR in vivo model [73]. The protective effects of caffeine are mediated by the adenosine A_2A_ receptor (A_2A_R) during the hyperoxic phase, while both A_2A_R-independent and -dependent mechanisms contribute during the hypoxic phase. Caffeine prevents neuronal cell death in the hyperoxic phase and reduces microglial cell activation as well as the expression of VEGF, TNF-α, and TGF-β1 in the hypoxic phase in OIR mice. Activation of microglial cells is known to accelerate endothelial cell death and abnormal vessel development. The administration of caffeine decreases both avascular and neovascular areas in OIR mice, highlighting its potential as a therapeutic agent for ROP.

Chlorogenic acid, a main compound in extracts of Aster koraiensis (AKE), a Korean herbal plant, was shown to reduce RNV in an OIR mouse model [74]. Chlorogenic acid inhibited tube formation in VEGF-induced vascular endothelial cells. The avascular area, neovascular area, and VEGF levels decreased in a dose-dependent manner following peritoneal administration of this compound. Although chlorogenic acid demonstrated anti-angiogenic effects, further studies are warranted to explore the underlying signaling pathways involved.

Combretastatin A4(CA-4), a stilbene isolated from *Combretum caffrum*, exhibits anti-angiogenic properties. Grigg et al. [75] reported that treatment with 80 ng/mL combretastatin suppresses cell proliferation, migration, and tube formation in HUVECs. Intraperitoneal injections of combretastatin dose-dependently reduce vascular angiogenesis in OIR mice without affecting normal retinal vessel development or causing vascular disruption such as hemorrhage or microthrombi. Compared to untreated mice, the mice group injected CA-4 at 3.124 mg/kg/day significantly inhibited RNV. CA-4 was well-tolerated with no significant side effects observed at a dose of 3.125 ng/kg/day. This study shows that CA-4 can suppress pathological RNV and serve as a potential therapy for proliferative retinopathy.

Deguelin, a rotenoid isolated from *Mundulea sericea*, also plays an inhibitory role in ROP. Kim et al. [76] reported significant reductions in retinal angiogenesis in mice injected with 0.1 μM intravitreal injection of deguelin compared with controls. Furthermore, intravitreal injections of deguelin at 0.1 μM, an effective therapeutic concentration, suppress HIF-1α and VEGF expression. HRECs administered with deguelin at concentrations ranging from 0 to 10 μM showed no significant changes in cell viability up to 1 μM, indicating absence of cytotoxicity. Intravitreal deguelin at 1 μM did not adversely affect physiological retinal development. Thus, this study demonstrates that deguelin can reduce pathological retinal angiogenesis without interrupting normal retinal vascular development or causing toxicity.

Genistein, an isoflavonoid derived from soybeans, has demonstrated properties that inhibit RNV [77]. Previously, it was reported to inhibit choriocapillaris regeneration and corneal neovascularization [78,79]. In the OIR in vivo model, intraperitoneal injection of 50–200 mg/kg/day of genistein suppressed the expression of VEGF and HIF-1α in a dose-dependent manner. Additionally, treatment with genistein reduced the number of nuclei protruding into the inner membrane of the mouse retina.

Honokiol, a biophenolic compound extracted from *Magnolia officinalis*, was found to reduce pathological RNV [80]. Expressions of HIF and VEGF were reduced in honokiol-treated ARPE-19 cells under hypoxia. Intraperitoneal injection of 10–20 mg/kg honokiol in OIR mice from P12 to P16 reduced avascular areas and neovascular tufts without interfering with physiological retinal vessel development, suggesting its inhibitory role in pathological retinal angiogenesis.

Luteolin, a flavone isolated from fruits and vegetables, was found to inhibit retinal angiogenesis in the OIR model. Park et al. [81] reported that luteolin reduced migration and tube formation in VEGF-stimulated HRMECs. Luteolin also suppressed ROS production and reduced the expression of VEGF in tertiary-butylhydroperoxide (t-BH)-stimulated HRMECs. Furthermore, luteolin inhibited the expression of HIF-1α and VEGF in hypoxia-induced human brain astrocytes. They also found that luteolin suppressed RNV in the ROP mouse model. This study showed that luteolin has anti-angiogenic and antioxidant properties and may be used to inhibit RNV. 

Omega-3 long-chain polyunsaturated fatty acids (LCPUFAs) derived from fish oil and omega-6 LCPUFAs derived from vegetable oil have shown protective effects against ROP [82,83]. Fu et al. reported that serum adiponectin (APN) levels were negatively correlated with ROP progression, while serum omega-3 LCPUFA levels were positively associated with serum APN levels in preterm infants [82]. Supplementation with omega-3 LCPUFA increases APN levels by inhibiting endoplasmic reticulum stress in adipocytes. The anti-angiogenic effect of omega-3 LUPUFA was reduced from 70% to 10% in APN knockout mice, suggesting that APN is critical for the efficacy of omega-3 LCPUFA. This study found that omega-3 supplementation in infants may reduce ROP development through regulating APN and RNV. Furthermore, Fu et al. investigated the effect of omega-3 and omega-6 LCPUFAs during phase I ROP mice model [83]. Dietary omega-3 LCPUFAs enhanced early vessel development in phase I ROP mice model. Additionally, single-cell transcriptomics revealed that omega-6 LCPUFAs were crucial for neuronal development and metabolism in the retina. The loss of APN reduced the effectiveness of both omega-3 and omega-6 LCPUFAs. Compared to mice administered omega-6 LCUPUFAs, mice fed with omega-3 LCUPUFAs had higher APN levels and increased expression of retinal APN receptors. This study builds upon and expands the finding of Fu et al. (2015) [82], providing a more detailed role of both omega-3 and -6 LCPUFAs in phase I and II of ROP.

Quercetin, a flavonoid found in many fruits and vegetables such as apples, berries, cabbage, and onions, exhibits anti-angiogenic properties [84]. Quercetin and 8MQPM, a premethylated form of quercetin, were shown to suppress cell viability, migration, and tube formation in retinoblastoma-conditioned medium-treated HRECs and inhibit vascular development in the aortic rings of rabbits. Additionally, quercetin and 8MQPM increased transendothelial electrical resistance (TEER), an indicator of blood-retinal barrier integrity. Both compounds effectively inhibited VEGFR2 downstream signaling pathways, including MEK/ERK, MEK/JNK, and PI3K/AKT, in conditioned medium-treated HRECs, demonstrating their anti-angiogenic properties.

Resveratrol, a stilbene extracted from red wine and grape skin, is another natural product that has demonstrated anti-angiogenic effects. Hu et al. recently reported that topical eye drops or intravitreal administration of resveratrol suppressed vascular permeability and vascular proliferation in a dose-dependent manner [85]. Additionally, resveratrol reduced the expression of CD31, an endothelial marker, and VEGF. It also alleviated oxidative stress in the retina by upregulating superoxide dismutase, a superoxide anion scavenging enzyme, and downregulating malondialdehyde, an oxidative stress marker.

**Table 1:** Natural compounds regulating ROP.

Natural Compounds	Effect of Eye	Cellular Model	Animal Model	Major Findings	References
2-Azahypoxanthine	Anti-angiogenic	CoCl2-induced ARPE-19 and 661 W cell line - 0.3–1000 μg/mL	OIR mice 300 mg/kg/day PO at P12-P16	- inhibits activation of HIF - reduce expression of VEGF level - Suppress RNV in OIR mice	[65]
Acetyl-11-keto-β-boswellic acid	Anti-angiogenic	VEGF-induced HRMECs - 1–10 μM	OIR mice - 5–20 mg/kg/day SC at P12-P16 or 50 μM IVT at P12 and P15	- inhibits proliferation, migration, and tube formation in VEGF-treated HRMECs - increases expression of SHP-1 and decreases the phosphorylation of STAT3 and VEGFR-2 in OIR mice	[66]
Apratoxin S4	Anti-angiogenic	VEGF-induced HRECs, human retinal pericyte - 1–25 nM	OIR mice - 0.0625, 0.125, 0.25 mg/kg IP at P12, 15 persistent RNV rabbit - 1.7 μM IVT 12 weeks after the first DL-AAA injection	- inhibits RNV through the downregulation of VEGF and Erk signaling pathways - reduces expression of PDGF-β in HRPCs	[69]
Astaxanthin	Anti-angiogenic, antiproliferative	N/A	Hyperoxia-induced retinopathy (HIR) mouse model: - 10 μg/mL and 100 μg/mL IVT at P12 - 0.5 mg/kg and 5 mg/kg IP at P12	- inhibit RNV in an HIR model - reduce mitochondria damage and apoptosis	[70]
Baicalin	Anti-angiogenic	N/A	OIR mice - 1, 10 mg/kg IP at P12-P17	- reduces the expression of MMP-2, MMP-9, angiotensin II, and VEGF - inhibits formation of avascular area and neovascular tufts and lumens	[71]
Caffeic acid	Anti-angiogenic antioxidant	VEGF-or H_2_O_2_- induced HRMECs - 10–200 μM	OIR mice - 100 μM IVT at P14	- suppresses cell proliferation, migration, and tube formation in VEGF-induced HRMECs and reduces ROS production and VEGF in H_2_O_2_-induced HRMECs - inhibits neovascular lumens	[72]
Caffeine	Anti-angiogenic	N/A	A_2A_R receptor and A_1_R knockout OIR mice - 0.1–1 g/L PO at P0-P17, P7-12, or P12-17 - 10 mg/kg IP at P7 and 2.5 mg/kg IP at P8-P16	- inhibits neuronal apoptosis at the hyperoxic phase through A_2A_R-dependent mechanism - suppresses microglial activation during the hypoxic phase through A_2A_R-dependent and independent mechanisms - reduces expression of VEGF, TNF-α, and TGF-β1 during the hypoxic phase	[73]
Chlorogenic acid	Anti-angiogenic	VEGF-induced HUVECs - 0.1–100 μg/mL	OIR mice - 25, 50 mg/kg/day IP at P12-P16	- suppresses VEGF-induced tube formation and RNV	[74]
Combretastatin-A4	Anti-angiogenic	N/A	OIR mice - 0.78–12 mg/kg/day IP at P12-P16	- inhibits proliferation, migration, and tube formation and induces apoptosis in ECs - reduces RNV in vivo	[75]
Deguelin	Anti-angiogenic	Hypoxia-induced HRECs - 0–10 μM	OIR mice - 0.1 μM IVT at P14	- reduces proliferation of HUVECs and RNV by suppressing HIF-1α and VEGF	[76]
Genistein	Anti-angiogenic	N/A	OIR mice - 50, 100, and 200 mg/kg/day IP at P14-P21	- inhibits RNV and expression of HIF-1α and VEGF in vivo	[77]
Honokiol	Anti-angiogenic	Hypoxia-induced human retinal pigment epithelial cells - 10–800 μM	OIR mice, - 10–20 mg/kg/day IP at P12-16	- inhibits progression ROP by downregulating HIF and VEGF signaling pathways and promotes physiological vascular development	[80]
Luteolin	Anti-angiogenic, anti-oxidative	VEGF or t-BH induced HRMECs 1–50 μM	OIR mice - 0.1–1 μM IVT at P14 (single dose)	- suppresses retinal angiogenesis and reduces VEGF-induced migration and tube formation and ROS in HRMECs	[81]
Omega-3 LCPFA	Anti-angiogenic	tunicamycin-induced 3T3-L1 adipocytes - 20 μL–50 mg/mL	Apn-knockout OIR mice - 2% omega-3 (1% DHA and 1% EPA) in diet at P1-17	- inhibit ER stress in adipose tissue - increase APN levels, subsequently reducing RNV	[82]
Omega-3 and -6 LCPFA	Omega-3: promote retinal vascular development Omega-6: essential for neuronal development and metabolism	N/A	Hyperglycemia-associated phase I ROP mice model (streptozocin-induced mice) - Omega-3 diet: 1% DHA + 1% EPA at P0-6 or 10 or P1-30 - Omega-6 diet: 2% AA at P0-6 or 10 or P1-30	- Omega-3 LCPUFAs enhance early vascular development in retina - Omega-6 LCPUFAs maintains retinal neuronal development and metabolism - Low APN levels are associated with reduced omega-3 and -6 LCUPUFA levels - Diet including omega-3 and -6 improves retinal function than diet including omega-3 alone	[83]
Quercetin	Anti-angiogenic	VEGF-A or CM-stimulated HRECs 25–100 μM	N/A	- inhibits RNV and cell viability and migration by downregulating VEGFR2 signaling pathway	[84]
Resveratrol	Anti-angiogenic, anti-oxidative	N/A	OIR mice 5, 25, and 50 mg/kg/day IVT or gtts twice a day at P12-P16	- reduces expression of VEGF and CD31 and oxidative damage	[85]

Abbreviations: AA, arachidonic acid; A_1_R, adenosine A1 receptor; A_2A_R, adenosine A2A receptor; ANG, angiogenin; APN, adiponectin; AREG, amphiregulin; cdc2, cell division cycle 2; AHX, 2-azahypoxanthine; CD31, cluster of differentiation 31; CM, conditioned medium; DHA, docosahexaenoic acid; DLL4, delta-like ligand 4; EC, endothelial cell; EPA, eicosapentaenoic acid; ER, endoplasmic reticulum; Erk, extracellular signal-regulated kinase; gtts, drops; HIF, hypoxia-inducible factor; HIR, hyperoxia-induced retinopathy; HREC, human retinal endothelial cell; HRMEC, human retinal microvascular endothelial cell; HRPC, human retinal pericyte; HUVEC, human umbilical vein endothelial cell; IGF, insulin-like growth factor-1; IP, intraperitoneal; IVT, intravitreal; LCPUFA, long-chain polyunsaturated fatty acid; MMP, matrix metalloproteinase; OIR, oxygen-induced retinopathy; PDGF-β, platelet-derived growth factor receptor beta; PEDF, pigment epithelium-derived factor; PO, by mouth; RNV, retinal neovascularization; ROP, retinopathy of prematurity; ROS, reactive oxygen species; SC, subcutaneous; SHP-1, Src homology region 2 domain-containing phosphatase 1; STAT3, signal transducer and activator of transcription 3; t-BH, tertiary-butylhydroperoxide; TGF-β1, transforming growth factor-beta 1; TNF-α, tumor necrosis factor alpha; VEGF, vascular endothelial growth factor.

## 4. Therapeutic Application of Natural Compounds in the Treatment of ROP

ROP is characterized by irregular vessel development in the retina, and its treatment aims to eliminate abnormal retinal vessel growth while preserving retinal function. Currently, laser photocoagulation and several anti-VEGF drugs are used to treat ROP [86,87]. Laser therapy using the argon or diode laser is considered the standard treatment for ROP. However, since the early 2000s, anti-VEGF agents have gained attention as pharmacologic therapy and have proven effective in treating ROP. Intravitreal anti-VEGF drugs, including bevacizumab, ranibizumab, and aflibercept, are used to treat ROP. Among these, aflibercept is the only drug currently approved for the treatment of ROP in the USA, Europe, Japan, and Brazil, while ranibizumab has been approved for ROP treatment in Europe [88,89]. Ranibizumab and bevacizumab target VEGF-A, whereas aflibercept inhibits a broader range, including VEGF-A, VEGF-B, and placental growth factor. Despite the availability of both non-pharmacologic and pharmacologic therapies for ROP, these treatments have limitations. For example, laser therapy carries a high risk of peripheral vision loss, scarring, and myopia. Conversely, intravitreal anti-VEGF inhibitors such as ranibizumab, bevacizumab, and aflibercept may cause systemic adverse effects in multiple organs in premature infants, which can be detrimental [1,90,91]. Given the limitations of current therapies, it is critical to explore novel therapeutic approaches.

Several natural compounds have been investigated in clinical trials for the treatment of ROP. Carotenoids, specifically L/Z, have been studied for their potential efficacy. According to a meta-analysis of three randomized controlled trials (RCTs), oral administration of L/Z did not reduce the incidence of ROP [92]. While two studies used a fixed dose, one study utilized a weight-based dose. Since there is no established target L/Z level in newborns and the optimal dosing is unknown, it is possible that the doses administered to newborns may be too low to reduce the incidence of ROP. Reports on the effects of polyunsaturated fatty acids have been inconclusive. Hellström et al. reported that enteral lipid supplementation reduces the risk of severe ROP [93]. However, a systematic review and meta-analysis of nine RCTs found that supplementation with LCPUFA did not reduce the incidence of ROP [94]. The differing results between Hellström et al. and the meta-analysis reported by Diggikar et al. may be attributed to differences in lipid formulation and sample size. While Hellström et al. used a specific 2:1 ratio of arachidonic acid (AA) to docosahexaenoic acid (DHA) and observed a significant reduction in severe ROP, the meta-analysis included studies with varying LCPUFA formulations, ratios, and doses. Since the effects of LCPUFA depend not only on the concentrations of AA and DHA but also on their ratio [95], future studies with optimal AA/DHA ratio supplementation and sufficient sample size to achieve statistical power may be needed to adequately assess the effect of LCPUFA. Conversely, early administration of caffeine in preterm infants within the first 3 days of birth was reported to reduce the risk of ROP compared to late administration after 3 days of birth in two systematic reviews and meta-analyses [96,97]. Caffeine may help to reduce hypoxia-induced VEGF signaling and inflammation and improve oxygenation and has neuroprotective effects [73,96,97]. Since caffeine is widely used in neonatal intensive care units for apnea of prematurity, it can serve as a preventive treatment for ROP in premature infants. While early caffeine supplementation has been shown to decrease the risk of ROP, future randomized controlled trials assessing the effects of caffeine to determine optimal administration time, dosing, and duration need to be conducted to establish its efficacy and optimize its use in the treatment of ROP.

Natural compounds derived from plants, animals, and microorganisms contain bioactive ingredients with therapeutic potential. These compounds can offer several advantages over conventional therapies, as they may possess diverse mechanisms of action, including anti-angiogenic, anti-inflammatory, antioxidant, and neuroprotective properties [98,99,100,101]. This multi-target approach can potentially provide comprehensive therapy for ROP. Given that premature infants are more vulnerable to systemic adverse effects and laser therapy can cause irreversible retinal damage, investigating natural compounds for ROP treatment is an attractive approach. Depending on the severity of ROP, natural compounds can be used as monotherapy or as an add-on therapy to reduce the dosage or frequency of conventional treatments. Furthermore, in-depth mechanistic studies are needed to elucidate the precise pathways regulating ROP pathogenesis.

## 5. Challenges and Strategies for Development of Natural Product-Based Drugs

Although natural products possess a wide variety of promising therapeutic potentials, several obstacles exist in developing natural product-based drugs. Regulatory concerns are particularly notable, as natural products in their original form may not be patentable [102]. Additionally, conflicts and discrepancies regarding the benefits derived from natural products may arise in the countries where the sources are collected [103]. Obtaining sufficient amounts of natural products for investigation can be challenging, especially if they originate from plants or marine sources in foreign countries. Furthermore, natural products derived from plants may have variable compositions depending on the location where the plants were obtained, resulting in inconsistencies [104]. The synthesis of natural products may be complicated, time-consuming, and costly [105]. Lastly, natural products may have poor oral bioavailability and instability [106].

**Figure 4:**
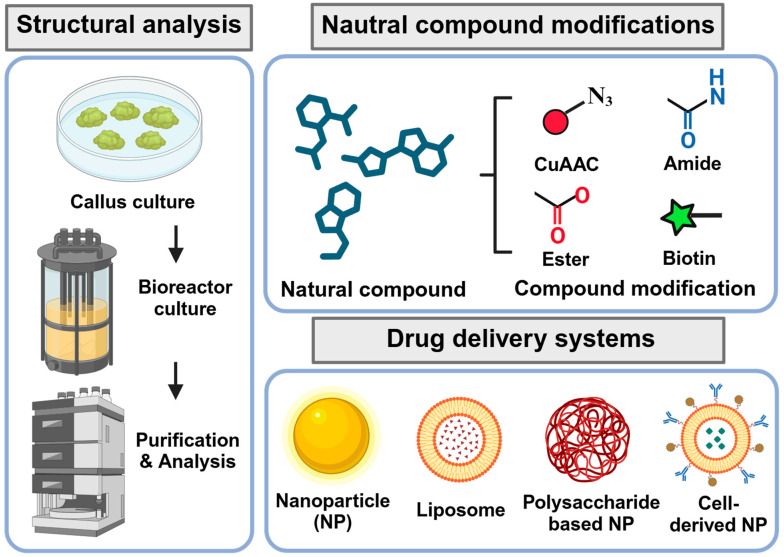
Strategies for the development of natural product-based drugs. Genetic engineering techniques, such as microbial fermentation or cell culture can be utilized to produce natural product-based drugs. The composition and consistency of active ingredients in natural product extracts can be verified through analytical methods, including mass spectrometry, nuclear magnetic resonance, and chromatography. Furthermore, modifying natural products can enhance their bioavailability, stability, or activity. The incorporation of drug delivery systems, such as nanoparticles or liposomes, can further improve the bioavailability and stability of natural products.

Several strategies can be considered to overcome these challenges (Figure 4). To acquire intellectual property rights such as patents, synthetic derivatives or analogs of natural products can be developed. Modifying original natural products can improve their bioavailability, stability, or activity. Countries sharing the sources of natural products can establish agreements, including regulations for benefit sharing, before investigating the natural products of interest. Access and procurement can be enhanced by cultivating plants or marine organisms under controlled environments or by utilizing genetic engineering techniques such as microbial fermentation or cell culture. Advanced analytical techniques, including mass spectrometry, nuclear magnetic resonance, and chromatography, along with rigorous quality control, can help standardize the composition of natural product extracts and verify consistency in the active ingredients [107]. Finally, the bioavailability and stability of natural products can be improved by developing novel drug delivery systems, such as nanoparticles or liposomes, or by modifying the chemical structures of natural products to enhance their pharmacokinetic profiles [106] (Figure 4). After developing natural product-based drugs, preclinical and clinical trials must be conducted to ensure the efficacy and safety of the novel drugs. 

## 6. Application of Advanced Technologies for Identification and Development of Natural Products

The identification of potential natural compounds for various diseases can be accelerated using screening techniques such as automated high-throughput screening (HTS), high-content screening (HCS), and mass spectrometry-based screening. Automated HTS enables the rapid identification of natural products with potential therapeutic effects by using automated robotic systems to screen thousands of natural compounds quickly [108]. Bioactivity, such as anti-angiogenic effects, can be detected using microplate readers and imaging systems. HCS combines HTS with automated microscopy and imaging analysis [109], allowing precise analysis of natural products at the cellular level, including the proliferation and migration of endothelial cells. Mass spectrometry-based screening is another method used to detect potential natural products for therapeutic use [110]. For example, matrix-assisted laser desorption/ionization time-of-flight mass spectrometry can identify natural compounds that may regulate ROP.

The efficacy and safety of natural compounds can be predicted using bioinformatics and computational modeling. Molecular docking can identify natural compounds with high binding affinity to receptor proteins and simulate their interactions [111]. This method can predict which natural products bind with key factors in ROP, such as VEGF, and anticipate their therapeutic potential. Quantitative structure–activity relationship models can screen large libraries of natural products, assess their chemical structures, and predict their bioactivity and safety [112]. Additionally, in silico computation tools predicting absorption, distribution, metabolism, excretion, and toxicity can be used to predict the bioavailability and toxicity of natural compounds [113].

The application of artificial intelligence (AI) in drug development is receiving increasing attention from researchers [114]. Machine learning (ML) and AI can assist in discovering novel natural products by screening potential targets through genome mining and the dereplication process. An ML algorithm can be applied to verify the structure of natural products. ML models, such as binary classifiers, can help to predict biological activities, absorption, distribution, metabolism, elimination profiles, and potential protein targets for natural products. Moreover, AI can assist in designing novel natural product-based drugs by applying deep learning generative models to optimize their chemical structures while maintaining biological activity [115].

Advanced in vitro models such as organ-on-chip systems, organoids, and microphysiological systems can serve as promising new approaches for assessing the therapeutic potential of natural products. Compared to traditional 2D cell culture, these advanced models provide a more physiologically relevant environment, allowing for accurate prediction of in vivo effects. Furthermore, multi-organ chip systems may help to evaluate the effects of natural products across tissues in various organs, enabling assessment of systemic response and metabolism [116]. Microfluidics, known as lab-on-chip technology, allows precise control over the cellular microenvironment by manipulating factors such as fluid flow and mechanical forces [116,117]. A retina-on-a-chip microfluidic device has been developed to deliver solutions through holes to the retina explant, facilitating local staining, drug administration, and response monitoring [118]. Additionally, a microfluidic chamber for retinal explant culture has been developed for optical stimulation and real-time monitoring [119]. Utilizing microfluidic technology enables evaluation of basic anatomy, physiology, and pathological processes of retinal tissue, as well as its detection and sensing capabilities.

The production of natural compounds can be increased by employing genetic engineering and synthetic biology. Using genetically modified microorganisms such as *E. coli* or yeast can enhance the yield of natural products [120]. Similarly, genetically modified plants can increase the biosynthesis of natural compounds [121]. Finally, programming entire biosynthetic pathways in microbes allows for the massive production of target natural compounds [122].

## 7. Conclusions

Natural products have potential as novel therapeutic agents for the treatment of ROP. Given the limitations of current ROP treatments, these products may present viable alternative options. Various natural compounds have demonstrated anti-angiogenic properties and effectiveness in mitigating ROP pathogenesis. However, their efficacy and safety must be verified through additional studies and clinical evaluations. The accumulating evidence, nonetheless, suggests that natural products could be promising alternatives for ROP treatment.

The development of drugs based on natural products is challenged by regulatory concerns, patent issues, source conflicts, and variable compositions. These challenges can be addressed by synthesizing derivatives that enhance the bioavailability and stability of natural products and by establishing international agreements regarding the sources of these products. Advances in genetic engineering have facilitated increased production of natural products, and the use of advanced analytical techniques ensures consistent composition. Moreover, recent innovations in screening and computational modeling permit large-scale screening of natural products to predict their therapeutic efficacy and safety. With technological advances in the identification and development of natural compounds, the discovery of natural products with significant therapeutic potential is likely to be expedited.

## References

[1] Ryu J. (2022). New Aspects on the Treatment of Retinopathy of Prematurity: Currently Available Therapies and Emerging Novel Therapeutics. Int. J. Mol. Sci..

[2] Hu X., Zhang J., Zhang M., Chen X., Han S., Zhu J. (2023). Incidence and Risk Factors for Retinopathy of Prematurity in a Tertiary Hospital in China. Clin. Ophthalmol..

[3] Nair A., El Ballushi R., Anklesaria B.Z., Kamali M., Talat M., Watts T. (2022). A Review on the Incidence and Related Risk Factors of Retinopathy of Prematurity Across Various Countries. Cureus.

[4] Yucel O.E., Eraydin B., Niyaz L., Terzi O. (2022). Incidence and risk factors for retinopathy of prematurity in premature, extremely low birth weight and extremely low gestational age infants. BMC Ophthalmol..

[5] Wang S., Liu J., Zhang X., Liu Y., Li J., Wang H., Luo X., Liu S., Liu L., Zhang J. (2024). Global, regional and national burden of retinopathy of prematurity among childhood and adolescent: A spatiotemporal analysis based on the Global Burden of Disease Study 2019. BMJ Paediatr. Open.

[6] Fieß A., Greven K., Mildenberger E., Urschitz M.S., Elflein H.M., Zepp F., Stoffelns B., Pfeiffer N., Schuster A.K. (2023). Visual acuity, amblyopia, and vision-related quality of life in preterm adults with and without ROP: Results from the Gutenberg prematurity eye study. Eye.

[7] Zhang L., Buonfiglio F., Fieß A., Pfeiffer N., Gericke A. (2024). Retinopathy of Prematurity—Targeting Hypoxic and Redox Signaling Pathways. Antioxidants.

[8] Sapieha P., Joyal J.S., Rivera J.C., Kermorvant-Duchemin E., Sennlaub F., Hardy P., Lachapelle P., Chemtob S. (2010). Retinopathy of prematurity: Understanding ischemic retinal vasculopathies at an extreme of life. J. Clin. Investig..

[9] Hartnett M.E. (2019). Discovering Mechanisms in the Changing and Diverse Pathology of Retinopathy of Prematurity: The Weisenfeld Award Lecture. Investig. Ophthalmol. Vis. Sci..

[10] Fevereiro-Martins M., Marques-Neves C., Guimarães H., Bicho M. (2023). Retinopathy of prematurity: A review of pathophysiology and signaling pathways. Surv. Ophthalmol..

[11] Chan E.C., van Wijngaarden P., Liu G.S., Jiang F., Peshavariya H., Dusting G.J. (2013). Involvement of Nox2 NADPH oxidase in retinal neovascularization. Investig. Ophthalmol. Vis. Sci..

[12] Wang H., Yang Z., Jiang Y., Hartnett M.E. (2014). Endothelial NADPH oxidase 4 mediates vascular endothelial growth factor receptor 2-induced intravitreal neovascularization in a rat model of retinopathy of prematurity. Mol. Vis..

[13] Poggi C., Giusti B., Vestri A., Pasquini E., Abbate R., Dani C. (2012). Genetic polymorphisms of antioxidant enzymes in preterm infants. J. Matern. Fetal Neonatal Med..

[14] Gu X., El-Remessy A.B., Brooks S.E., Al-Shabrawey M., Tsai N.T., Caldwell R.B. (2003). Hyperoxia induces retinal vascular endothelial cell apoptosis through formation of peroxynitrite. Am. J. Physiol. Cell Physiol..

[15] Kim H., Kim J., Ryu J. (2022). Noncoding RNAs as a novel approach to target retinopathy of prematurity. Front. Pharmacol..

[16] Hartnett M.E., Penn J.S. (2012). Mechanisms and management of retinopathy of prematurity. N. Engl. J. Med..

[17] Krock B.L., Skuli N., Simon M.C. (2011). Hypoxia-induced angiogenesis: Good and evil. Genes. Cancer.

[18] Stone J., Itin A., Alon T., Pe’er J., Gnessin H., Chan-Ling T., Keshet E. (1995). Development of retinal vasculature is mediated by hypoxia-induced vascular endothelial growth factor (VEGF) expression by neuroglia. J. Neurosci..

[19] Ramshekar A., Hartnett M.E. (2021). Vascular Endothelial Growth Factor Signaling in Models of Oxygen-Induced Retinopathy: Insights Into Mechanisms of Pathology in Retinopathy of Prematurity. Front. Pediatr..

[20] Hartnett M.E., Martiniuk D., Byfield G., Geisen P., Zeng G., Bautch V.L. (2008). Neutralizing VEGF decreases tortuosity and alters endothelial cell division orientation in arterioles and veins in a rat model of ROP: Relevance to plus disease. Investig. Ophthalmol. Vis. Sci..

[21] Rathi S., Jalali S., Patnaik S., Shahulhameed S., Musada G.R., Balakrishnan D., Rani P.K., Kekunnaya R., Chhablani P.P., Swain S. (2017). Abnormal Complement Activation and Inflammation in the Pathogenesis of Retinopathy of Prematurity. Front. Immunol..

[22] Sivakumar V., Foulds W.S., Luu C.D., Ling E.A., Kaur C. (2011). Retinal ganglion cell death is induced by microglia derived pro-inflammatory cytokines in the hypoxic neonatal retina. J. Pathol..

[23] Smith L.E. (2005). IGF-1 and retinopathy of prematurity in the preterm infant. Biol. Neonate.

[24] Ribatti D., Presta M., Vacca A., Ria R., Giuliani R., Dell’Era P., Nico B., Roncali L., Dammacco F. (1999). Human erythropoietin induces a pro-angiogenic phenotype in cultured endothelial cells and stimulates neovascularization in vivo. Blood.

[25] Takagi H., Koyama S., Seike H., Oh H., Otani A., Matsumura M., Honda Y. (2003). Potential role of the angiopoietin/tie2 system in ischemia-induced retinal neovascularization. Investig. Ophthalmol. Vis. Sci..

[26] Pugh C.W., Ratcliffe P.J. (2003). Regulation of angiogenesis by hypoxia: Role of the HIF system. Nat. Med..

[27] Apte R.S., Chen D.S., Ferrara N. (2019). VEGF in Signaling and Disease: Beyond Discovery and Development. Cell.

[28] Wu P.-Y., Fu Y.-K., Lien R.-I., Chiang M.-C., Lee C.-C., Chen H.-C., Hsueh Y.-J., Chen K.-J., Wang N.-K., Liu L. (2023). Systemic Cytokines in Retinopathy of Prematurity. J. Pers. Med..

[29] Zhu H., Zhang S. (2018). Hypoxia inducible factor-1α/vascular endothelial growth factor signaling activation correlates with response to radiotherapy and its inhibition reduces hypoxia-induced angiogenesis in lung cancer. J. Cell Biochem..

[30] Song Y.Y., Liang D., Liu D.K., Lin L., Zhang L., Yang W.Q. (2023). The role of the ERK signaling pathway in promoting angiogenesis for treating ischemic diseases. Front. Cell Dev. Biol..

[31] Hoxhaj G., Manning B.D. (2020). The PI3K-AKT network at the interface of oncogenic signalling and cancer metabolism. Nat. Rev. Cancer.

[32] Lee M.Y., Luciano A.K., Ackah E., Rodriguez-Vita J., Bancroft T.A., Eichmann A., Simons M., Kyriakides T.R., Morales-Ruiz M., Sessa W.C. (2014). Endothelial Akt1 mediates angiogenesis by phosphorylating multiple angiogenic substrates. Proc. Natl. Acad. Sci. USA.

[33] Phung T.L., Ziv K., Dabydeen D., Eyiah-Mensah G., Riveros M., Perruzzi C., Sun J., Monahan-Earley R.A., Shiojima I., Nagy J.A. (2006). Pathological angiogenesis is induced by sustained Akt signaling and inhibited by rapamycin. Cancer Cell.

[34] Demain A.L., Sanchez S. (2009). Microbial drug discovery: 80 years of progress. J. Antibiot..

[35] Miner J., Hoffhines A. (2007). The discovery of aspirin’s antithrombotic effects. Tex. Heart Inst. J..

[36] Mashour N.H., Lin G.I., Frishman W.H. (1998). Herbal Medicine for the Treatment of Cardiovascular Disease: Clinical Considerations. Arch. Intern. Med..

[37] Schultz C. (2014). Safety and efficacy of cyclosporine in the treatment of chronic dry eye. Ophthalmol. Eye Dis..

[38] Di L. (2015). Strategic approaches to optimizing peptide ADME properties. AAPS J..

[39] Huang G., Lv M., Hu J., Huang K., Xu H. (2016). Glycosylation and Activities of Natural Products. Mini Rev. Med. Chem..

[40] Patridge E., Gareiss P., Kinch M.S., Hoyer D. (2016). An analysis of FDA-approved drugs: Natural products and their derivatives. Drug Discov. Today.

[41] Chordia P., MacArthur R.D. (2013). Crofelemer, a novel agent for treatment of non-infectious diarrhea in HIV-infected persons. Expert. Rev. Gastroenterol. Hepatol..

[42] Jung W., Choi H., Kim J., Kim J., Kim W., Nurkolis F., Kim B. (2023). Effects of natural products on polycystic ovary syndrome: From traditional medicine to modern drug discovery. Heliyon.

[43] Li C., Xu Y., Zhang J., Zhang Y., He W., Ju J., Wu Y., Wang Y. (2023). The effect of resveratrol, curcumin and quercetin combination on immuno-suppression of tumor microenvironment for breast tumor-bearing mice. Sci. Rep..

[44] Hu H.C., Lei Y.H., Zhang W.H., Luo X.Q. (2022). Antioxidant and Anti-inflammatory Properties of Resveratrol in Diabetic Nephropathy: A Systematic Review and Meta-analysis of Animal Studies. Front. Pharmacol..

[45] Koushki M., Amiri-Dashatan N., Ahmadi N., Abbaszadeh H.A., Rezaei-Tavirani M. (2018). Resveratrol: A miraculous natural compound for diseases treatment. Food Sci. Nutr..

[46] Yang J., Miao X., Yang F.J., Cao J.F., Liu X., Fu J.L., Su G.F. (2021). Therapeutic potential of curcumin in diabetic retinopathy (Review). Int. J. Mol. Med..

[47] Kaabi Y.A. (2022). Potential Roles of Anti-Inflammatory Plant-Derived Bioactive Compounds Targeting Inflammation in Microvascular Complications of Diabetes. Molecules.

[48] Nathan J., Shameera R., Devarajan N., Perumal E. (2023). Role of berberine on angiogenesis and blood flow hemodynamics using zebrafish model. J. Appl. Toxicol..

[49] Maci S., Santos R. (2015). The beneficial role of lutein and zeaxanthin in cataracts. Nutrafoods.

[50] Mrowicka M., Mrowicki J., Kucharska E., Majsterek I. (2022). Lutein and Zeaxanthin and Their Roles in Age-Related Macular Degeneration-Neurodegenerative Disease. Nutrients.

[51] Keenan T.D.L., Agron E., Keane P.A., Domalpally A., Chew E.Y., Areds, Groups A.R. (2024). Oral Antioxidant and Lutein/Zeaxanthin Supplements Slow Geographic Atrophy Progression to the Fovea in Age-Related Macular Degeneration. Ophthalmology.

[52] Alugoju P., Krishna Swamy V.K.D., Anthikapalli N.V.A., Tencomnao T. (2023). Health benefits of astaxanthin against age-related diseases of multiple organs: A comprehensive review. Crit. Rev. Food Sci. Nutr..

[53] Giannaccare G., Pellegrini M., Senni C., Bernabei F., Scorcia V., Cicero A.F.G. (2020). Clinical Applications of Astaxanthin in the Treatment of Ocular Diseases: Emerging Insights. Mar. Drugs.

[54] Shoda C., Miwa Y., Nimura K., Okamoto K., Yamagami S., Tsubota K., Kurihara T. (2020). Hypoxia-Inducible Factor Inhibitors Derived from Marine Products Suppress a Murine Model of Neovascular Retinopathy. Nutrients.

[55] Shoda C., Lee D., Miwa Y., Yamagami S., Nakashizuka H., Nimura K., Okamoto K., Kawagishi H., Negishi K., Kurihara T. (2024). Inhibition of hypoxia-inducible factors suppresses subretinal fibrosis. FASEB J..

[56] Kang J.M., Lin S. (2018). Ginkgo biloba and its potential role in glaucoma. Curr. Opin. Ophthalmol..

[57] Castro-Castaneda C.R., Altamirano-Lamarque F., Ortega-Macías A.G., Santa Cruz-Pavlovich F.J., Gonzalez-De la Rosa A., Armendariz-Borunda J., Santos A., Navarro-Partida J. (2022). Nutraceuticals: A Promising Therapeutic Approach in Ophthalmology. Nutrients.

[58] Wang D., Chen Y., Li J., Wu E., Tang T., Singla R.K., Shen B., Zhang M. (2024). Natural Products for the Treatment of Age-Related Macular Degeneration. Phytomedicine.

[59] Fanaro G.B., Marques M.R., Calaza K.D.C., Brito R., Pessoni A.M., Mendonça H.R., Lemos D.E.A., de Brito Alves J.L., de Souza E.L., Cavalcanti Neto M.P. (2023). New Insights on Dietary Polyphenols for the Management of Oxidative Stress and Neuroinflammation in Diabetic Retinopathy. Antioxidants.

[60] Wang N., Zhang C., Xu Y., Tan H.Y., Chen H., Feng Y. (2021). Berberine improves insulin-induced diabetic retinopathy through exclusively suppressing Akt/mTOR-mediated HIF-1α/VEGF activation in retina endothelial cells. Int. J. Biol. Sci..

[61] Lou Y., Oberpriller J.C., Carlson E.C. (1997). Effect of hypoxia on the proliferation of retinal microvessel endothelial cells in culture. Anat. Rec..

[62] Pierce E.A., Foley E.D., Smith L.E.H. (1996). Regulation of Vascular Endothelial Growth Factor by Oxygen in a Model of Retinopathy of Prematurity. Arch. Ophthalmol..

[63] Li Y., Busoy J.M., Zaman B.A.A., Tan Q.S.W., Tan G.S.W., Barathi V.A., Cheung N., Wei J.J., Hunziker W., Hong W. (2018). A novel model of persistent retinal neovascularization for the development of sustained anti-VEGF therapies. Exp. Eye Res..

[64] Smith L.E., Wesolowski E., McLellan A., Kostyk S.K., D’Amato R., Sullivan R., D’Amore P.A. (1994). Oxygen-induced retinopathy in the mouse. Investig. Ophthalmol. Vis. Sci..

[65] Lee D., Miwa Y., Wu J., Shoda C., Jeong H., Kawagishi H., Tsubota K., Kurihara T. (2020). A Fairy Chemical Suppresses Retinal Angiogenesis as a HIF Inhibitor. Biomolecules.

[66] Lulli M., Cammalleri M., Fornaciari I., Casini G., Dal Monte M. (2015). Acetyl-11-keto-β-boswellic acid reduces retinal angiogenesis in a mouse model of oxygen-induced retinopathy. Exp. Eye Res..

[67] Lim S., Lee K.W., Kim J.Y., Kim K.D. (2024). Consideration of SHP-1 as a Molecular Target for Tumor Therapy. Int. J. Mol. Sci..

[68] Chen Z., Han Z.C. (2008). STAT3: A critical transcription activator in angiogenesis. Med. Res. Rev..

[69] Qiu B., Tan A., Veluchamy A.B., Li Y., Murray H., Cheng W., Liu C., Busoy J.M., Chen Q.Y., Sistla S. (2019). Apratoxin S4 Inspired by a Marine Natural Product, a New Treatment Option for Ocular Angiogenic Diseases. Investig. Ophthalmol. Vis. Sci..

[70] Küçüködük A., Helvacioglu F., Haberal N., Dagdeviren A., Bacanli D., Yilmaz G., Akkoyun I. (2019). Antiproliferative and anti-apoptotic effect of astaxanthin in an oxygen-induced retinopathy mouse model. Can. J. Ophthalmol..

[71] Jo H., Jung S.H., Yim H.B., Lee S.J., Kang K.D. (2015). The effect of baicalin in a mouse model of retinopathy of prematurity. BMB Rep..

[72] Kim J.H., Lee B.J., Kim J.H., Yu Y.S., Kim K.W. (2009). Anti-angiogenic effect of caffeic acid on retinal neovascularization. Vasc. Pharmacol..

[73] Zhang S., Zhou R., Li B., Li H., Wang Y., Gu X., Tang L., Wang C., Zhong D., Ge Y. (2017). Caffeine preferentially protects against oxygen-induced retinopathy. FASEB J..

[74] Kim J., Lee Y.M., Jung W., Park S.B., Kim C.S., Kim J.S. (2018). Aster koraiensis Extract and Chlorogenic Acid Inhibit Retinal Angiogenesis in a Mouse Model of Oxygen-Induced Retinopathy. Evid. Based Complement. Altern. Med..

[75] Griggs J., Skepper J.N., Smith G.A., Brindle K.M., Metcalfe J.C., Hesketh R. (2002). Inhibition of proliferative retinopathy by the anti-vascular agent combretastatin-A4. Am. J. Pathol..

[76] Kim J.H., Kim J.H., Yu Y.S., Shin J.Y., Lee H.Y., Kim K.W. (2008). Deguelin inhibits retinal neovascularization by down-regulation of HIF-1alpha in oxygen-induced retinopathy. J. Cell Mol. Med..

[77] Wang B., Zou Y., Li H., Yan H., Pan J.S., Yuan Z.L. (2005). Genistein inhibited retinal neovascularization and expression of vascular endothelial growth factor and hypoxia inducible factor 1alpha in a mouse model of oxygen-induced retinopathy. J. Ocul. Pharmacol. Ther..

[78] Majji A.B., Hayashi A., Kim H.C., Grebe R.R., de Juan E. (1999). Inhibition of choriocapillaris regeneration with genistein. Investig. Ophthalmol. Vis. Sci..

[79] Joussen A.M., Rohrschneider K., Reichling J., Kirchhof B., Kruse F.E. (2000). Treatment of corneal neovascularization with dietary isoflavonoids and flavonoids. Exp. Eye Res..

[80] Vavilala D.T., O’Bryhim B.E., Ponnaluri V.K., White R.S., Radel J., Symons R.C., Mukherji M. (2013). Honokiol inhibits pathological retinal neovascularization in oxygen-induced retinopathy mouse model. Biochem. Biophys. Res. Commun..

[81] Park S.W., Cho C.S., Jun H.O., Ryu N.H., Kim J.H., Yu Y.S., Kim J.S., Kim J.H. (2012). Anti-angiogenic effect of luteolin on retinal neovascularization via blockade of reactive oxygen species production. Investig. Ophthalmol. Vis. Sci..

[82] Fu Z., Lofqvist C.A., Shao Z., Sun Y., Joyal J.S., Hurst C.G., Cui R.Z., Evans L.P., Tian K., SanGiovanni J.P. (2015). Dietary omega-3 polyunsaturated fatty acids decrease retinal neovascularization by adipose-endoplasmic reticulum stress reduction to increase adiponectin. Am. J. Clin. Nutr..

[83] Fu Z., Yan W., Chen C.T., Nilsson A.K., Bull E., Allen W., Yang J., Ko M., SanGiovanni J.P., Akula J.D. (2022). Omega-3/Omega-6 Long-Chain Fatty Acid Imbalance in Phase I Retinopathy of Prematurity. Nutrients.

[84] Lupo G., Cambria M.T., Olivieri M., Rocco C., Caporarello N., Longo A., Zanghì G., Salmeri M., Foti M.C., Anfuso C.D. (2019). Anti-angiogenic effect of quercetin and its 8-methyl pentamethyl ether derivative in human microvascular endothelial cells. J. Cell Mol. Med..

[85] Hu W.H., Zhang X.Y., Leung K.W., Duan R., Dong T.T., Qin Q.W., Tsim K.W. (2022). Resveratrol, an Inhibitor Binding to VEGF, Restores the Pathology of Abnormal Angiogenesis in Retinopathy of Prematurity (ROP) in Mice: Application by Intravitreal and Topical Instillation. Int. J. Mol. Sci..

[86] Pertl L., Steinwender G., Mayer C., Hausberger S., Poschl E.M., Wackernagel W., Wedrich A., El-Shabrawi Y., Haas A. (2015). A Systematic Review and Meta-Analysis on the Safety of Vascular Endothelial Growth Factor (VEGF) Inhibitors for the Treatment of Retinopathy of Prematurity. PLoS ONE.

[87] Hartnett M.E., Stahl A. (2023). Laser versus Anti-VEGF: A Paradigm Shift for Treatment-Warranted Retinopathy of Prematurity. Ophthalmol. Ther..

[88] Raghuveer T.S., Zackula R.E., Hartnett M.E. (2024). Aflibercept to treat retinopathy of prematurity: Need for more research. J. Perinatol..

[89] Stahl A., Azuma N., Wu W.-C., Lepore D., Sukgen E., Nakanishi H., Mazela J., Leal S., Pieper A., Schlief S. (2024). Systemic exposure to aflibercept after intravitreal injection in premature neonates with retinopathy of prematurity: Results from the FIREFLEYE randomized phase 3 study. Eye.

[90] Heo J.I., Ryu J. (2024). Exosomal noncoding RNA: A potential therapy for retinal vascular diseases. Mol. Ther. Nucleic Acids.

[91] Dogra M.R., Vinekar A. (2023). Role of Anti-Vascular Endothelial Growth Factor (Anti-VEGF) in the Treatment of Retinopathy of Prematurity: A Narrative Review in the Context of Middle-Income Countries. Pediatr. Health Med. Ther..

[92] Cota F., Costa S., Giannantonio C., Purcaro V., Catenazzi P., Vento G. (2022). Lutein supplementation and retinopathy of prematurity: A meta-analysis. J. Matern. Fetal Neonatal Med..

[93] Hellström A., Nilsson A.K., Wackernagel D., Pivodic A., Vanpee M., Sjöbom U., Hellgren G., Hallberg B., Domellöf M., Klevebro S. (2021). Effect of Enteral Lipid Supplement on Severe Retinopathy of Prematurity: A Randomized Clinical Trial. JAMA Pediatr..

[94] Diggikar S., Aradhya A.S., Swamy R.S., Namachivayam A., Chandrasekaran M. (2022). Effect of Enteral Long-Chain Polyunsaturated Fatty Acids on Retinopathy of Prematurity: A Systematic Review and Meta-Analysis. Neonatology.

[95] Najm S., Lofqvist C., Hellgren G., Engstrom E., Lundgren P., Hard A.L., Lapillonne A., Savman K., Nilsson A.K., Andersson M.X. (2017). Effects of a lipid emulsion containing fish oil on polyunsaturated fatty acid profiles, growth and morbidities in extremely premature infants: A randomized controlled trial. Clin. Nutr. ESPEN.

[96] Karlinski Vizentin V., Madeira de Sá Pacheco I., Fahel Vilas Bôas Azevêdo T., Florêncio de Mesquita C., Alvim Pereira R. (2024). Early versus Late Caffeine Therapy Administration in Preterm Neonates: An Updated Systematic Review and Meta-Analysis. Neonatology.

[97] Kua K.P., Lee S.W. (2017). Systematic review and meta-analysis of clinical outcomes of early caffeine therapy in preterm neonates. Br. J. Clin. Pharmacol..

[98] Riaz M., Khalid R., Afzal M., Anjum F., Fatima H., Zia S., Rasool G., Egbuna C., Mtewa A.G., Uche C.Z. (2023). Phytobioactive compounds as therapeutic agents for human diseases: A review. Food Sci. Nutr..

[99] Pangestuti R., Kim S.-K. (2011). Biological activities and health benefit effects of natural pigments derived from marine algae. J. Funct. Foods.

[100] Pereira L., Cotas J. (2023). Therapeutic Potential of Polyphenols and Other Micronutrients of Marine Origin. Mar. Drugs.

[101] Lançon A., Frazzi R., Latruffe N. (2016). Anti-oxidant, anti-inflammatory and anti-angiogenic properties of resveratrol in ocular diseases. Molecules.

[102] Harrison C. (2014). Patenting natural products just got harder. Nat. Biotechnol..

[103] Lee H., Son J., Min S., Lee H., Park M.S. (2023). Natural Resources Conflicts on Borderlands by the Five Spheres of Earth System. Land.

[104] Kellogg J.J., Paine M.F., McCune J.S., Oberlies N.H., Cech N.B. (2019). Selection and characterization of botanical natural products for research studies: A NaPDI center recommended approach. Nat. Prod. Rep..

[105] Morikawa T., Tamura S., Wang T. (2020). Editorial: Discovery and Total Synthesis of Bio-functional Natural Products From Traditional Medicinal Plants. Front. Chem..

[106] Sachdeva A., Dhawan D., Jain G.K., Yerer M.B., Collignon T.E., Tewari D., Bishayee A. (2022). Novel Strategies for the Bioavailability Augmentation and Efficacy Improvement of Natural Products in Oral Cancer. Cancers.

[107] Wang H., Chen Y., Wang L., Liu Q., Yang S., Wang C. (2023). Advancing herbal medicine: Enhancing product quality and safety through robust quality control practices. Front. Pharmacol..

[108] Ayon N.J. (2023). High-Throughput Screening of Natural Product and Synthetic Molecule Libraries for Antibacterial Drug Discovery. Metabolites.

[109] Fraietta I., Gasparri F. (2016). The development of high-content screening (HCS) technology and its importance to drug discovery. Expert. Opin. Drug Discov..

[110] Dueñas M.E., Peltier-Heap R.E., Leveridge M., Annan R.S., Büttner F.H., Trost M. (2023). Advances in high-throughput mass spectrometry in drug discovery. EMBO Mol. Med..

[111] Agu P.C., Afiukwa C.A., Orji O.U., Ezeh E.M., Ofoke I.H., Ogbu C.O., Ugwuja E.I., Aja P.M. (2023). Molecular docking as a tool for the discovery of molecular targets of nutraceuticals in diseases management. Sci. Rep..

[112] Dara S., Dhamercherla S., Jadav S.S., Babu C.M., Ahsan M.J. (2022). Machine Learning in Drug Discovery: A Review. Artif. Intell. Rev..

[113] Simoben C.V., Babiaka S.B., Moumbock A.F.A., Namba-Nzanguim C.T., Eni D.B., Medina-Franco J.L., Günther S., Ntie-Kang F., Sippl W. (2023). Challenges in natural product-based drug discovery assisted with in silico-based methods. RSC Adv..

[114] Paul D., Sanap G., Shenoy S., Kalyane D., Kalia K., Tekade R.K. (2021). Artificial intelligence in drug discovery and development. Drug Discov. Today.

[115] Saldívar-González F.I., Aldas-Bulos V.D., Medina-Franco J.L., Plisson F. (2022). Natural product drug discovery in the artificial intelligence era. Chem. Sci..

[116] Wu Q., Liu J., Wang X., Feng L., Wu J., Zhu X., Wen W., Gong X. (2020). Organ-on-a-chip: Recent breakthroughs and future prospects. Biomed. Eng. Online.

[117] Horowitz L.F., Rodriguez A.D., Ray T., Folch A. (2020). Microfluidics for interrogating live intact tissues. Microsyst. Nanoeng..

[118] Dodson K.H., Echevarria F.D., Li D., Sappington R.M., Edd J.F. (2015). Retina-on-a-chip: A microfluidic platform for point access signaling studies. Biomed. Microdevices.

[119] Alarautalahti V., Ragauskas S., Hakkarainen J.J., Uusitalo-Järvinen H., Uusitalo H., Hyttinen J., Kalesnykas G., Nymark S. (2019). Viability of Mouse Retinal Explant Cultures Assessed by Preservation of Functionality and Morphology. Investig. Opthalmology Vis. Sci..

[120] Pham J.V., Yilma M.A., Feliz A., Majid M.T., Maffetone N., Walker J.R., Kim E., Cho H.J., Reynolds J.M., Song M.C. (2019). A Review of the Microbial Production of Bioactive Natural Products and Biologics. Front. Microbiol..

[121] Hirschi K.D. (2020). Genetically Modified Plants: Nutritious, Sustainable, yet Underrated. J. Nutr..

[122] Zhu X., Liu X., Liu T., Wang Y., Ahmed N., Li Z., Jiang H. (2021). Synthetic biology of plant natural products: From pathway elucidation to engineered biosynthesis in plant cells. Plant Commun..

